# Evaluation of a Shotgun Metagenomics Approach for Detection of ESBL- and/or Carbapenemase-Producing *Enterobacterales* in Culture Negative Patients Recovered from Acute Leukemia

**DOI:** 10.3390/microorganisms11020402

**Published:** 2023-02-04

**Authors:** Pilar Lumbreras-Iglesias, Carlos Sabater, Ainhoa Fernández Moreno, Paula López de Ugarriza, Ana Fernández-Verdugo, Abelardo Margolles, María Rosario Rodicio, Teresa Bernal, Javier Fernández

**Affiliations:** 1 Traslational Microbiology Group, Instituto de Investigación Sanitaria del Principado de Asturias (ISPA), 33011 Oviedo, Spain; 2 Department of Clinical Microbiology, Hospital Universitario Central de Asturias (HUCA), 33011 Oviedo, Spain; 3 Dairy Research Institute of Asturias (IPLA), Spanish National Research Council (CSIC), 33300 Villaviciosa, Spain; 4 MicroHealth Group, Instituto de Investigación Sanitaria del Principado de Asturias (ISPA), 33011 Oviedo, Spain; 5 Department of Hematology, Hospital Universitario Central de Asturias (HUCA), Instituto de Investigación Sanitaria del Principado de Asturias (ISPA), Instituto de Oncología del Principado de Asturias (IUOPA), 33011 Oviedo, Spain; 6 Department of Functional Biology, Microbiology Area, University of Oviedo, 33006 Oviedo, Spain; 7 Research & Innovation, Artificial Intelligence and Statistical Department, Pragmatech AI Solutions, 33001 Oviedo, Spain; 8 Centro de Investigación Biomédica en Red-Enfermedades Respiratorias, 28029 Madrid, Spain

**Keywords:** acute leukemia, metagenomics, ESBL, carbapenemase, *Enterobacterales*

## Abstract

Patients diagnosed with acute leukemia (AL) have a weakened immune system. Infections acquired by these patients are cause for concern and especially worrisome when Gram-negative multidrug-resistant (MDR) bacteria are involved, as they are difficult to treat, especially in the case of ESBL- and/or carbapenemase-producing *Enterobacterales*. Culture-based approaches have been relied on over the past decades as the method of choice for the early detection of gut colonization by MDR Gram-negative bacteria. However, various studies have indicated its limited sensitivity, underlining the need for new screening procedures in onco-hematological patients. Here, we evaluated a shotgun metagenomics approach to detect ESBL- and/or carbapenemase-producing *Enterobacterales* in the gut of 28 patients who had recovered from AL, which were previously colonized by these bacteria but cured at the time of sampling, as judged by culture-based methods. No ESBL or carbapenemase determinants were detected among the many resistance genes found by the metagenomics approach, supporting that patients were truly decolonized, with considerable consequences for their future clinical management. Due to the relatively low number of patients available for the present investigation, further studies should be conducted to support the utility and applicability of metagenomics for the routine screening of MDR bacteria in onco-hematological patients.

## 1. Introduction

Blood cancers such as acute leukemia (AL) are malignant disorders of the blood and bone marrow. Patients with AL have a weakened immune system that makes them vulnerable to bacterial infections [1]. The disease can be treated by chemotherapy or hematopoietic stem cell transplantation (HSCT), sometimes both, which are aggressive procedures that destroy stem cells, cause neutropenia [2], and further diminish the defensive capacity of the immune system [2]. Aggravating the problem, patients diagnosed with AL face numerous risk factors for the colonization/infection by multidrug resistant (MDR) bacteria. In addition to profound neutropenia, these factors include damage to the gastrointestinal mucosa and continued exposure to broad-spectrum antibiotics and to the hospital environment [3,4].

In particular, infections caused by Gram-negative MDR bacteria are frequent and particularly worrisome in these patients, since they increase the infectious mortality between two and four times compared to infections due to susceptible bacteria, especially when the antimicrobial therapy administered is not adequate [5,6]. Among the resistant bacteria, *Enterobacterales* producing extended spectrum beta-lactamases (ESBLs) or carbapenemases are of concern, since the therapeutic options for their treatment are limited [3,5].

Surveillance culture-based methods have demonstrated to be effective for the early detection of colonized patients by ESBL- and/or carbapenemase-producing *Enterobacterales* and are routinely performed in many hospitals, mainly in intensive care units and hematology wards [7,8,9]. This screening allows taking infection control measures rapidly, but also to adjust proper empirical antimicrobial regimes, once the infection develops. Thus, both overtreatment in non-colonized patients and undertreatment in colonized patients can be avoided, situations which are generally associated with an increase of infectious mortality [9]. Despite its usefulness, some studies found that culture-based methods are not sensitive enough for the detection of all cases of colonization by MDR Gram-negative bacteria [7,10,11].

In the last years, metagenomics sequencing has been introduced in clinical microbiology laboratories. It will potentially revolutionize the diagnosis of infectious diseases, aiding traditional culture-based microbiology, among other things due its broad diagnostic spectrum and its high sensitivity [12].

The aim of the present work was to evaluate the usefulness of a shotgun metagenomics approach to detect ESBL- and/or carbapenemase-producing *Enterobacterales* in patients who had recovered from AL, and who had been previously colonized by them, but currently decolonized according to the last rectal swab or fecal culture.

## 2. Materials and Methods

A total of 21 rectal swabs and 7 fecal samples from 28 outpatients recovered from AL or high-risk myelodysplastic syndrome treated with intensive chemotherapy were collected. The patients were followed-up in a tertiary Spanish hospital, the “Hospital Universitario Central de Asturias” (HUCA). Screening was performed weekly during the active phase of the disease. Once the disease remised, patients were followed up in consultations approximately every month, and a rectal swab was taken at these visits. All of them had been colonized in the gut by ESBL- and/or carbapenemase-producing *Enterobacterales* before sample collection but decolonized at the time of sampling according to the last culture results. An additional rectal swab of a patient with a relapse of acute leukemia and currently colonized by an ESBL-producing *Escherichia coli* (harboring a *bla*_CTX-M-15_ gene) was used as a positive control.

Screening of ESBL- and/or carbapenemase-producing *Enterobacterales* was performed in all patients using three different methods. (i) Samples were first plated on ChromID ESBL and ChromID CARBA SMART (bioMérieux, Marcy l’Etoile, France) and incubated at 37 ºC for 48 h. Whenever bacterial growth was observed, the colony identification was performed by MALDI-TOF/MS (Bruker Daltonics, Billerica, MA, USA) and antimicrobial susceptibility was determined by the Microscan system (Beckman Coulter, Brea, CA, USA) and interpreted according to current EUCAST guidelines (www.eucast.org, accessed on 1 December 2022). (ii) After DNA extraction performed directly from samples in a MagCore device (RBC Bioscience, New Taipei City, Taiwan), ESBL- and carbapenemase-encoding genes were screened using the AMR Direct Flow Chip Kit (Vitro, Sevilla, Spain), a microarray multiplex PCR-based method including the most common ESBL- and carbapenemase-encoding resistance genes found in Gram-negative bacteria [13]. (iii) Lastly, a metagenomics approach was performed, for which DNA was extracted from the samples with the QIAamp Fast DNA Stool Mini Kit (Qiagen, Hilden, Germany) and sequenced in a NovaSeq 6000 platform with Illumina technology, to generate 150 bp paired-end reads. TORMES v1.3.0 [14], a script which implements a comprehensive pipeline for metagenomics analysis (including quality control of the reads, de novo genome assembly, and screening of antimicrobial resistance genes, among others), was applied to all samples. Quality filtering was accomplished by Prinseq v0.20.4 [15]. Reads with a quality score lower than 25 or with less than 125 bp were excluded from the analysis. The assembly of the filtered reads was performed both with SPAdes v3.15.2 [16] and MEGAHIT v1.2.9 [17], and identification of resistance genes was carried out with ResFinder [18], ARG-ANNOT [19] and CARD [20] databases, using a minimum percent of identity and coverage of 80%.

## 3. Results

Demographics and clinical information about the evaluated patients are summarized in Table 1. The clinical diagnosis was acute myeloid leukemia in ~90% of patients (25/28), with the remaining three suffering from acute lymphoblastic leukemia (2) and myelodysplastic syndrome (1). The median time elapsed between the study sample and the last chemotherapy cycle administered was 849 days (interquartile range 564–1290) while the median time since the last detected colonization to sample collection was 798 days (interquartile range 522–1268). Bacterial culture did not detect ESBL- or carbapenemase-producing *Enterobacterales* in the samples under study, and the AMR Flow Chip did not detect their coding genes. In the control sample, however, culture results revealed the presence of an *E. coli* strain expressing the ESBL phenotype, which was positive for *bla*_CTX-M_ according to the multiplex PCR assay. Metagenomics analysis identified the *bla*_CTX-M-15_ gene in the positive control, but neither ESBL- nor carbapenemase-encoding genes were found in any of the remaining samples (Table 1).

Two of the samples failed to be assembled with SPAdes but were successfully assembled with MEGAHIT, which assembled all. Many other resistance genes were detected (neither coding for ESBLs nor carbapenemases) within the three resistance gene identification databases in the metagenomics analysis of samples from all patients. ResFinder results and clinical information about the outpatients studied are also summarized in Table 1. No differences were observed whether the assembly was achieved with SPAdes or MEGAHIT. This result supports the reliability of the analysis. Clean reads of the samples successfully assembled with SPAdes and MEGAHIT generated an average of 651,397 and 241,810 contigs per sample, respectively, with a GC content of 47% in both cases. The average numbers of assembled reads per sample were 25,686,378 and 25,779,802 for SPAdes and MEGAHIT, respectively, which had an average read length of 149 bp. Median sequencing depth was 22X with MEGAHIT and 16X with SPAdes.

## 4. Discussion

In this work, we tried to answer the question whether metagenomics sequencing is more sensitive than conventional techniques for rectal/fecal sample screening of MDR bacteria in onco-hematological patients. This has important implications from both the microbiological and the clinical point of view. Regarding the former, clinical microbiological laboratories are interested in knowing if they may miss colonizations by these bacteria in their routine workflows. As stated in the introduction, although culture-based methods constitute the gold standard and the more extended method for the fecal/rectal screening of MDR bacteria in most laboratories, their low sensitivity when compared to molecular techniques is well documented [21,22,23]. Regarding molecular methods based on PCR, although they are generally more sensitive, they have also limitations, since they include only a limited number of targets that do not represent the total diversity of ESBLs or carbapenemases already reported and potentially carried by *Enterobacterales* [24]. Not many published studies have actually evaluated the suitability of metagenomics sequencing for the detection of fecal carriage of MDR *Enterobacterales*. In one of these previous studies aimed at the fecal occurrence of MDR bacteria in children, both ESBL- and carbapenemase-encoding genes were detected in several patients with negative cultures [22]. In fact, culture-based methods as the gold standard to evaluate advanced molecular methods such as next-generation sequencing is imperfect according to several works [12,22]. By contrast, in the present study, there were no conflicting results between the conventional methods and the shotgun metagenomics approach. This could be owed to the fact that the patients were truly decolonized, or that the sensitivity of the metagenomics approach was not superior to those of the culture-based method and the multiplex PCR. Since the higher sensitivity of metagenomics sequencing in comparison with traditional culture methods was demonstrated in previous studies [12,22], the first hypothesis is more plausible, which affects the clinical implications of the present study. Regarding the patients, they had not been exposed to chemotherapy or antibiotics for a median of 28 months, which may have contributed to the restoration of the gut microbiome. There are several implications of gut decolonization that may impact the clinical outcomes of patients. First, it is well known that more than half of the patients with acute leukemia will experience a relapse of their disease [25]. Relapse is commonly treated with the administration of further chemotherapy, followed by allogeneic transplant, followed by a period of hospitalization, mucosal damage, neutropenia, and antibiotic exposure. If the patients were colonized by ESBL- and/or carbapenemase-producing *Enterobacterales* (even in a low relative abundance), all the aforementioned factors would exert a selective pressure that ultimately would end in an increased risk of bacteremia [12,26,27]. Second, under-detection of colonization could favor the spread of resistant bacteria to other onco-hematological patients at risk, because early contacts will not be prevented. This is particularly relevant in the case of carbapenem-resistant *K. pneumoniae* isolates, considering that they are mainly spread by hospital transmission [28].

We are aware that the current work has certain limitations, such as the low number of patients studied and the variability in terms of the time elapsed between the last colonization and the moment of sampling. Some studies based on culture methods have analyzed how long colonization by ESBL- and carbapenemase-producing *Enterobacterales* lasts, obtaining very different results. For instance, Mo et al. observed that the mean duration of carbapenemase-producing *Enterobacterales* carriage was 86 days and that the probability of decolonization after one year was 98.5% [29]. However, Zimmerman et al., analyzing a cohort of 137 subjects colonized by these bacteria, reported that the mean time to culture-negativity was 387 days, with 39% of the patients still colonized in the year follow-up [30]. The duration of colonization can be affected by many factors, such as exposure to the healthcare system or antibiotics [29,30], and to the best of our knowledge it has not been specifically studied in the case of hematological patients. Because of this, we decided to analyze patients at very different times since their last detected colonization. Another limitation of our study derives from the indistinct use of fecal samples or rectal swabs, since it could generate bias when comparing results. Although it could be argued that rectal swabs are not representative of the whole gut, recent studies have demonstrated that they can be used as a surrogate of fecal samples for metagenomics studies, being nearly equivalent [31,32]. In addition, rectal swabs are the currently recommended and most used specimen for the screening of MDR bacteria in the gut [11].

Shotgun metagenomics sequencing has great potential for the diagnosis and management of infections in patients with hematologic malignancies, both for MDR bacterial screening and for etiological diagnosis. For instance, a recent study demonstrated that this approach increased the detection of relevant viruses or bacteria in the blood of patients with febrile neutropenia that were missed by conventional methods [33]. However, it must be mentioned that the new techniques are more time consuming and expensive, and the identification of the isolate’s species is more complex than with the culture method. Metagenomics techniques have additional limitations as they are not yet well standardized, they require complex bioinformatic analyses, as well as very good quality of the reads in order to obtain a high coverage and perform a correct assembly and annotation [34]. Yet, metagenomics approaches have been improved over the last years and are now becoming cheaper and less time consuming, which is crucial for AL patients [22].

## 5. Conclusions

Metagenomics shotgun sequencing could potentially be used as a complement to standard cultures and PCR-based methods for the screening of MDR bacteria. However, standardization and simplification of the procedures are essential for the implementation of this approach in the routine workflows of clinical microbiology laboratories. In contrast to other studies, the metagenomics study presented here indicated that patients recovered from AL who have a negative culture for ESBL- and/or carbapenemase-producing *Enterobacterales*, but were previously colonized by them, were indeed decolonized, which may have important repercussions for the future clinical management of the patients. However, more studies with higher numbers of patients are needed not only to confirm this result, but also to analyze the utility and applicability of metagenomics for the routine screening of MDR bacteria in patients with hematological malignancies.

## Figures and Tables

**Table 1:** Clinical and microbiological information of outpatients recovered from acute leukemia or that underwent stem cell transplantation and who had been colonized by ESBL- and/or carbapenemase-producing *Enterobacterales*.

Patient	Age/ Gender	Underlying Disease/ Status	Sample	Previous ESBL- and/or Carbapenemase-Producing *Enterobacterales* Gut Colonization	Time from Last Gut Colonization (days)/Time from Administration of the Last Cycle of Chemotherapy (Days)	Current Detection of ESBL- and/or Carbapenemase-Producing *Enterobacterales* by Culture/PCR/Metagenomic Shotgun Sequencing	Other Antibiotic Resistant Genes Detected by Metagenomic Approach
SMet_1	53/M	AML/CR	Feces	*Klebsiella pneumoniae* ESBL	1047/1293	No	*cfxA5*, *cepA*, *ant(3″)-Ia*, *ant(6)-Ia*, *aph(3′)-III*, *tet*(Q), *tet*(W), *tet*(X), *tet*(40), *catS*, *erm*(B), *erm*(F), *erm*(G), *mef*(A), *msr*(D), *dfrA1*
SMet_2	57/F	AML/CR	Rectal swab	*Enterobacter cloacae* ESBL + OXA-48	542/537	No	*bla*_TEM-1A_, *bla*_ACI-1_, *cfxA3*, *ant(6)-Ia*, *aph(3′)-III*, *tet*(M), *tet*(O), *tet*(Q), *tet*(W), *erm*(A), *erm*(F)
SMet_3	59/M	AML/CR	Rectal swab	*K. pneumoniae* ESBL	1246/1215	No	*cfxA6*, *sul2*, *tet*(Q), *tet*(W), *lnu*(C)
SMet_4	69/F	AML/CR	Rectal swab	*Citrobacter freundii* OXA-48	790/795	No	*ant(6)-Ia*, *aph(3′)-III*, *tet*(O), *tet*(Q), *tet*(W), *tet*(40), *cat*, *erm*(B)
**SMet_5 ^1^**	**70/F**	**AML/Relapse**	**Rectal swab**	***Escherichia coli* ESBL**	**0/537**	**Yes**	***bla*_CTX-M-15_, *cfxA3*, *bla*_OXA-1_, *aac(6′)-Ib-cr*, *erm*(F), *mph*(A), *dfrA17***
SMet_6	47/F	AML/CR	Rectal swab	*E. coli* ESBL + OXA-48	433/477	No	*bla*_Z_, *cfxA4*, *cepA*, *bla*_OXA-85_, *aac(6′)-II*, *ant(6)-Ia*, *ant(6)-Ib*, *aph(3′)-III*, *aph(3″)-Ib*, *tet*B(46), *tet*(M), *tet*(Q), *tet*(T), *tet*(W), *tet*(X), *tet*(40), *erm*(A), *erm*(B), *erm*(F), *lnu*(C), *lsa*(A), *mef*(A), *mph*(C), *msr*(C), *msr*(D), *dfrG*, *fosB*
SMet_7	74/F	AML/CR	Rectal swab	*E. cloacae* ESBL + OXA-48	806/834	No	*cfxA5*, *ant(6)-Ia*, *aph(3′)-III*, *tet*(M), *tet*(O), *tet*(W), *tet*(X), *tet*(40), *cat*, *erm*(A), *erm*(B), *erm*(F), *erm*(G), *mdf*(A), *mef*(A), *msr*(D)
SMet_8	47/F	AML/CR	Rectal swab	*K. pneumoniae* ESBL	578/588	No	*bla*_TEM-1C_, *cfxA5*, *sul2*, *ant(6)-Ia*, *aph(3′)-III*, *aph(3″)-Ib*, *aph(6)-Id*, *tet*(M), *tet*(Q), *tet*(W), *floR*, *erm*(A), *erm*(B), *erm*(F), *erm*(G), *erm*(X), *erm*(X), *lnu*(C), *lsa*(C), *mdf*(A)
SMet_9	31/M	AML/CR	Rectal swab	*K. pneumoniae* ESBL	1464/1359	No	*cfxA3*, *cfxA6*, *ant(6)-Ia*, *aph(3′)-III*, *tet*(O), *tet*(Q), *tet*(Q), *tet*(W), *tet*(X), *tet*(32), *erm*(A), *erm*(B), *erm*(F), *erm*(G), *erm*(X), *mef*(A), *msr*(D)
SMet_10	46/F	AML/CR	Rectal swab	*K. pneumoniae* ESBL	1365/1377	No	*bla*_TEM-1C_, *bla*_ACI-1_, *cfxA3*, *sul2*, *aph(3′)-Ia*, *aph(3″)-Ib*, *aph(6)-Id*, *tet*(B), *tet*(O), *tet*(Q), *tet*(Q), *tet*(X), *floR*, *erm*(F), *mdf*(A), *mef*(A), *mph*(A), *msr*(D), *dfrA14*
SMet_11	70/M	AML/CR	Rectal swab	*K. pneumoniae* ESBL	1375/1263	No	*bla*_TEM-1B_, *cfxA3*, *sul2*, *aph(3″)-Ib*, *aph(6)-Id*, *tet*(A), *tet*(M), *tet*(Q), *mdf*(A), *qnrB19*, *dfrA5*
SMet_12	39/F	AML/CR	Rectal swab	*E. coli* ESBL	728/867	No	*cfxA3*, *ant(6)-Ia*, *aph(3′)-III*, *tet*(M), *tet*(Q), *tet*(X), *erm*(A), *erm*(F), *mdf*(A), *msr*(D)
SMet_13	56/F	AML/CR	Rectal swab	*K. pneumoniae* ESBL	267/729	No	*bla*_TEM-1B_, *cfxA3*, *sul2*, *sul3*, *aadA2*, *ant(3″)-Ia*, *ant(6)-Ia*, *aph(3′)-III*, *aph(3″)-Ib*, *aph(6)-Id*, *tet*(A), *tet*(O), *tet*(Q), *tet*(W), *tet*(X), *tet*(32), *tet*(40), *catP*, *cmlA1*, *floR*, *erm*(B), *erm*(F), *erm*(X), *lnu*(C), *mdf*(A), *mef*(B), *dfrA1*, *dfrA12*
SMet_14	50/F	AML/CR	Rectal swab	*E. coli* OXA-48	333/351	No	*cfxA3*, *tet*(O), *tet*(Q), *erm*(A), *erm*(F), *lsa*(C)
SMet_15	49/F	AML/CR	Rectal swab	*K. pneumoniae* ESBL	1211/1098	No	*cfxA5*, *ant(3″)-Ia*, *ant(6)-Ia*, *aph(3′)-III*, *tet*(Q), *tet*(W), *tet*(40), *cat*, *erm*(A), *erm*(G), *erm*(X), *mdf*(A), *mef*(A), *msr*(D)
SMet_16	43/M	AML/CR	Rectal swab	*K. pneumoniae* ESBL	224/405	No	*bla*_TEM-1B_, *cfxA3*, *sul3*, *ant(3″)-Ia*, *ant(6)-Ia*, *aph(3′)-III*, *aph(3″)-Ib*, *aph(6)-Id*, *tet*(A), *tet*(O), *tet*(Q), *tet*(W), *tet*(X), *tet*(32), *tet*(40), *cmx*, *erm*(A), *erm*(B), *erm*(F), *erm*(X), *lnu*(C), *mdf*(A), *dfrA1*
SMet_17	68/F	AML/CR	Rectal swab	*K. pneumoniae* ESBL	1230/1419	No	*cfxA5*, *sul2*, *aac(6′)-Im*, *ant(6)-Ia*, *aph(2″)-Ib*, *aph(2″)-Ig*, *aph(3′)-III*, *aph(3″)-Ib*, *aph(6)-Id*, *tet*(W), *tet*(40), *erm*(F)
SMet_18	51/M	MDS/CR	Rectal swab	*K. pneumoniae* ESBL	720/630	No	*bla*_TEM-1B_, *cfxA3*, *cfxA5*, *bla*_DHA-14_, *bla*_OXA-347_, *sul1*, *sul2*, *aac(3)-IId*, *aac(3)-XI*, *aadA2*, *aph(3′)-Ia*, *aph(3′)-III*, *aph(3″)-Ib*, *aph(6)-Id*, *tet*(B), *tet*(M), *tet*(O), *tet*(Q), *tet*(X), *tet*(32), *tet*(40), *catA1*, *cmx*, *erm*(A), *erm*(B), *erm*(F), *erm*(X), *lnu*(C), *mph*(A), *dfrA12*
SMet_19	61/F	AML/CR	Rectal swab	*K. pneumoniae* ESBL	987/831	No	*bla*_TEM-1B_, *cfxA5*, *sul2*, *ant(3″)-Ia*, *ant(6)-Ia*, *aph(3′)-III*, *aph(3″)-Ib*, tet(M), *tet*(Q), *tet*(W), *tet*(X), *catS*, *erm*(B), *erm*(F), *erm*(G), *lnu*(C), *mdf*(A), *dfrA1*, *dfrA14*
SMet_20	58/M	ALL/CR	Rectal swab	*K. pneumoniae* ESBL	1218	No	*cfxA3*, *sul1*, *sul2*, *ant(3″)-Ia*, *ant(6)-Ia*, *aph(3′)-III*, *aph(3″)-Ib*, *aph(6)-Id*, *tet*(C), *tet*(O), *tet*(Q), *tet*(W), *erm*(B), *lnu*(C), *mdf*(A), *dfrA1*
SMet_21	39/F	ALL/CR	Rectal swab	*E. coli* ESBL	85/51	No	*bla*_TEM-1B_, *cfxA3*, *cfxA5*, *sul2*, *ant(6)-Ia*, *aph(3′)-Ia*, *aph(3′)-III*, *aph(6)-Id*, *tet*(A), *tet*(M), *tet*(O), *tet*(Q), *tet*(Q), *tet*(X), *erm*(A), *erm*(B), *erm*(X), *lnu*(C), *mef*(A), *mph*(A), *dfrA14*
SMet_22	67/M	AML/CR	Rectal swab	*K. pneumoniae* OXA-48	715/864	No	*bla*_SHV-145_, *tet*(M), *erm*(B), *lsa*(A), *oqxA*, *oqxB*, *fosA*
SMet_23	72/F	AML/CR	Rectal swab	*K. pneumoniae* ESBL	288/303	No	*cfxA3*, *blaDHA-1*, *sul1*, *ant(6)-Ia*, *aph(3′)-III*, *tet*(O), *tet*(Q), *tet*(W), *tet*(X), *cat*, *erm*(A), *erm*(B), *erm*(F), *mdf*(A), *mph*(A), *qnrB4*
SMet_24	54/M	AML/CR	Feces	*K. pneumoniae* ESBL	1362/1398	No	*cfxA3*, *cepA*, *sul2*, *aac(6′)-Im*, *ant(3″)-Ia*, *ant(6)-Ia*, *aph(3″)-Ib*, *aph(6)-Id*, *tet*(M), *tet*(O), *tet*(W), *tet*(32), *catP*, *erm*(B), *erm*(F), *erm*(G), *lnu*(C)
SMet_25	65/F	AML/CR	Feces	*K. pneumoniae* ESBL	612/588	No	*cfxA3*, *cepA*, *sul2*, *ant(6)-Ia*, *aph(3′)-III*, *aph(3″)-Ib*, *aph(6)-Id*, *tet*(M), *tet*(O), *tet*(Q), *tet*(X), *tet*(32), *tet*(40), *cat*, *erm*(B), *erm*(F), *erm*(G), *lnu*(C), *lnu*(C), *mef*(A)
SMet_26	57/F	AML/CR	Feces	*K. pneumoniae* ESBL	1356/1314	No	*cfxA3*, *cepA*, *sul2*, *ant(6)-Ia*, *aph(3′)-III*, *aph(6)-Id*, *tet*(Q), *tet*(X), *tet*(40), *cat*, *catS*, *erm*(B), *erm*(F), *erm*(G), *lnu*(C), *mef*(A)
SMet_28	60/F	AML/CR	Feces	*K. pneumoniae* ESBL	1471/1428	No	*bla*_TEM-1B_, *cfxA5*, *cfxA6*, *sul2*, *ant(3″)-Ia*, *ant(6)-Ia*, *aph(3′)-III*, *aph(3″)-Ib*, *aph(6)-Id*, *tet*(A), *tet*(B), *tet*(O), *tet*(Q), *tet*(W), *tet*(X), *tet*(40), *tet*(44), *cat*, *catS*, *erm*(B), *erm*(F), *erm*(G), *lnu*(C), *mdf*(A), *mef*(A), *msr*(D), *dfrA1*
SMet_29	69/F	AML/CR	Feces	*K. pneumoniae* ESBL	1334/1341	No	*cfxA3*, *cfxA6, ant(6)-Ia*, *tet*(Q), *tet*(W), *tet*(X), *tet*(32), *tet*(40), *cat, catP*, *erm*(B), *erm*(F), *erm*(G), *lnu*(C), *mef*(A), *msr*(D)
SMet_30	48/M	AML/CR	Feces	*K. pneumoniae* ESBL	463/411	No	*cfxA4*, *sul1*, *aadA5*, *ant(6)-Ia*, *tet*(A), *tet*(O), *tet*(Q), *tet*(W), *tet*(X), *cat*, *erm*(F), *erm*(G), *lnu*(C), *mdf*(A), *mef*(A), *mph*(A), *msr*(D), *dfrA17*

ESBL, extended spectrum beta-lactamase; M, male; F, female; AML, acute myeloid leukemia; MDS, myelodysplastic syndrome; ALL, acute lymphoblastic leukemia; CR, complete remission. ^1^ This sample was used as positive control, an *E. coli* expressing an ESBL phenotype was detected by culture, and PCR and metagenomic shotgun approach revealed that it carried the *bla*_CTX-M-15_.

## Data Availability

The raw sequences data were deposited in the Sequence Read Archive (SRA) of the NCBI (https://www.ncbi.nlm.nih.gov/sra, accessed on 20 December 2022) under BioProject code PRJNA914091, with BioSample codes from SAMN32318268 to SAMN32318297.
